# The Coronavirus Health and Impact Survey (CRISIS) reveals reproducible correlates of pandemic-related mood states across the Atlantic

**DOI:** 10.1038/s41598-021-87270-3

**Published:** 2021-04-14

**Authors:** Aki Nikolaidis, Diana Paksarian, Lindsay Alexander, Jacob Derosa, Julia Dunn, Dylan M. Nielson, Irene Droney, Minji Kang, Ioanna Douka, Evelyn Bromet, Michael Milham, Argyris Stringaris, Kathleen R. Merikangas

**Affiliations:** 1 Center for the Developing Brain, The Child Mind Institute, New York, NY USA; 2 Genetic Epidemiology Research Branch, Intramural Research Program, National Institute of Mental Health, Bethesda, MD USA; 3 Section On Clinical and Computational Psychiatry (CompΨ), National Institute of Mental Health, National Institutes of Health, Bethesda, MD USA; 4 Department of Psychiatry, Renaissance School of Medicine at Stony, Brook University, Stony Brook, NY USA; 5 Nathan Kline Institute for Psychiatric Research, Orangeburg, NY USA; 6 Johns Hopkins Bloomberg School of Public Health, Baltimore, MD USA

**Keywords:** Epidemiology, Paediatric research, Human behaviour

## Abstract

The COVID-19 pandemic and its social and economic consequences have had adverse impacts on physical and mental health worldwide and exposed all segments of the population to protracted uncertainty and daily disruptions. The CoRonavIruS health and Impact Survey (CRISIS) was developed for use as an easy to implement and robust questionnaire covering key domains relevant to mental distress and resilience during the pandemic. Ongoing studies using CRISIS include international studies of COVID-related ill health conducted during different phases of the pandemic and follow-up studies of cohorts characterized before the COVID pandemic. In the current work, we demonstrate the feasibility, psychometric structure, and construct validity of this survey. We then show that pre-existing mood states, perceived COVID risk, and lifestyle changes are strongly associated with negative mood states during the pandemic in population samples of adults and in parents reporting on their children in the US and UK. These findings are highly reproducible and we find a high degree of consistency in the power of these factors to predict mental health during the pandemic.

## Introduction

Since its first documented occurrence in December 2019, COVID-19 has taken an enormous toll on human life and health and is in line to become the leading cause of death in many countries, including the US. Along with the immediate health impacts, the virus and prevention strategies have perturbed the core structure of daily life, including financial security, work, school, recreation, and social interactions. The COVID-19 pandemic stands in stark contrast to recent epidemics such as SARS and MERS in terms of the total number of cases and deaths^1^. Prior mass disasters (World Trade Center attacks, mass shootings), natural disasters (hurricanes, floods) and environmental exposures (oil spills, radiation exposures) have been associated with increases in depression, posttraumatic stress disorder (PTSD), substance use, generalized anxiety disorder and a range of other mental health outcomes^2–6^. These studies were conducted primarily in the aftermath of these catastrophes. Much less is known about the risk and protective factors for well-being during and after prolonged threats^3^, like the COVID-19 pandemic, which continues to unfold.

The pernicious mental health effects of the COVID-19 pandemic may result from death of loved ones, disease severity, social isolation and quarantine, unemployment, financial hardship, domestic violence, and educational disruptions^7,8^. Each of these factors is independently associated with psychological comorbidities^9–15^. Apart from studies of the neuropsychiatric impact of COVID-19 on health care workers and people who contracted the virus^11,16–18^, there are a growing number of international longitudinal surveys designed to document community-level pandemic-related psychological distress. Many of these surveys have been adapted to encompass a wider range of mental health outcomes than measured in previous epidemics^4^, with the most common domains including stress, anxiety, loneliness, depression, social support, media and technology use, sleep, and post-traumatic stress. Most of these surveys included established symptom scales, such as the Generalized Anxiety Disorder (GAD-7), Patient Health Questionnaire (PHQ-9), and UCLA Loneliness Scale^16,19,20^, as documented by the COVID-MINDS network of longitudinal studies on the global mental health impact of COVID-19 (www.COVIDminds.org/). Published findings from these studies have shown high levels of anxiety and depression symptoms post COVID-19 based on cut-points from US and European sources. However, the most robust risk factors for disaster-related mental ill health—prior psychopathology and exposure severity^2,6^—remain largely understudied to date. With some exceptions^21–27^ few of the COVID-19 specific assessment tools developed to track mental health responses to the pandemic have been psychometrically validated.

Understanding prolonged threats like the COVID-19 pandemic necessitates the implementation of instruments that measure the risk and protective factors for well-being that are both psychometrically validated and statistically reliable. Aligning with NIH COVID-19 research priorities^28,29^, the CoRonavIruS health and Impact Survey (CRISIS) Initiative was established as a collaborative and multidisciplinary effort to identify predictors of acute and long-term psychopathology. Key predictors include impairment and disability associated with the COVID-19 pandemic in samples that were well-characterized prior to the pandemic across the globe. The first step was to develop, pilot, and test the psychometric properties of a comprehensive instrument that captured a core set of domains. Specifically, the CRISIS instrument was designed to assess pertinent mental, behavioral, and physical health domains that capture the multi-level emotional and behavioral impact of the pandemic, as well as a range of pandemic-related and pre-existing risk and protective factors. The CRISIS includes forms for adults ages 19–64, parent reports for children aged 9–18, and youth aged 9–18. The following domains are assessed: (1) background and demographic characteristics, including household composition and crowding; (2) physical and mental health 3 months prior to the pandemic; (3) COVID-19 exposure and infection status; (4) life changes due to the pandemic; (5) concerns and worries associated with COVID-19; (6) current well-being determined by the circumplex model of affect^30,31^; and (7) behavioral factors, such as media use, sleep, physical activity, and substance use. We also developed a short form of the CRISIS for follow up of the samples that excludes the background and the three month prior physical and mental health sections. Both the baseline and follow up surveys are licensed on Creative Commons (CC) BY4.0 and are available for download at crisissurvey.org.

This article describes the properties of the CRISIS in relatively large (n = 5646) pilot samples of adults and parents in the United States (US) and United Kingdom (UK) collected in April 2020. Across the multiple samples, the aims were to: (1) describe the CRISIS and assess its acceptability and feasibility, (2) evaluate the factor structure of the major domains; (3) examine the test–retest reliability and construct validity of these domains across the multiple samples, and (4) estimate the relative importance of the measured domains to current mood states (operationalized as mood over the previous 2 weeks).

Our primary hypotheses were that (1) the Mood States and COVID worries constructs would show strong unidimensional characteristics and would replicate these characteristics across all samples in the US and UK; and (2) Prior mental health status and COVID worries would be the strongest predictors of current mental health. Recognizing the sustained impact of the pandemic on lifestyle (e.g., social distancing, financial insecurity) and behaviors (e.g., sleep, exercise), we explored these domains to identify additional factors that would have predictive value.

## Methods

### Samples

Pilot data were collected between April 7th and 17th, 2020, through Prolific Academic (https://www.prolific.ac/) (PA), an online crowdsourced survey recruitment service. Participants who signed up to join the PA participant pool received monetary compensation for their time. PA participants have been shown to be more diverse and provide higher quality data than similar data collection platforms^32^. We requested four samples of 1500 participants from Prolific Academic, from the US and UK, both adult self report and parent report. We wanted to prioritize countries with high exposure to COVID-19 at the time. Portions of the sample were targeted at regions that were more severely impacted by COVID-19 in late March 2020 (New York, California, London, and Manchester). Based on Prolific Academic’s guidelines, representative samples between 300 and 1500 participants were possible through their platform. We chose to acquire data only through the two countries that PA offers representative sampling to both maximize the generalizability and reproducibility of our findings. For parent reports, users were screened based on having a child between 5 and 17 years old, and reported on their oldest child in that age range. We opted to restrict ourselves to adult self report and parent report samples and not acquire child self report due to difficulties in obtaining child report data in these samples at the time of acquisition. No additional exclusion criteria were given to PA.

We received a total of 5928 unduplicated responses, from which we dropped 282 with incomplete forms. The final analytic sample sizes were 1527 US adults (231 California; 246 New York), 1539 UK adults (248 London; 238 Manchester), 1121 US parents (27 California; 19 New York), and 1459 UK parents (172 London; 219 Manchester). Samples were further divided into training (2/3) and hold-out (1/3) samples for assessing the reproducibility of associations with current mood states. Resulting training data sample sizes were 935 (US Adult), 938 (UK Adult), 673 (US Parent), and 877 (UK Parent). Separate 24-h test–retest reliability samples were obtained from 74 US adults, 76 UK adults, 71 US parents, and 75 UK parents concurrently with the main US/UK samples. Sample demographics are summarized in Table 1. Missing data are summarized in Supplemental Table 1.

**Table 1:** Frequencies and percentages of key demographic variables and COVID-related experiences by sample.

	Adult US	Adult UK	Parent US	Parent UK
	935 N (%)	938 N (%)	673 N (%)	877 N (%)
**Sex**				
Male	397 (42.9)	403 (43.5)	356 (53.6)	460 (52.8)
Female	521 (56.3)	523 (56.4)	305 (45.9)	410 (47.0)
Other	8 (0.9)	1 (0.1)	3 (0.5)	2 (0.2)
**Age**				
Under 30	396 (42.4)	289 (30.8)	83 (12.4)	89 (10.2)
30–49	322 (34.5)	395 (42.1)	548 (81.5)	722 (82.6)
50 and over	216 (23.1)	254 (27.1)	41 (6.1)	63 (7.2)
**Child age**				
5 and Under	**	**	96 (14.3)	158 (18.1)
6–13	**	**	413 (61.5)	507 (57.9)
14–17	**	**	144 (21.4)	183 (20.9)
18 and over	**	**	19 (2.8)	27 (3.1)
**Race**				
Asian	128 (13.7)	65 (6.9)	21 (3.1)	41 (4.7)
Black	78 (8.3)	41 (4.4)	39 (5.8)	41 (4.7)
Hispanic	148 (15.8)	19 (2.0)	97 (14.4)	15 (1.7)
White	530 (56.7)	770 (82.1)	484 (71.9)	755 (86.1)
Other	51 (5.5)	43 (4.6)	32 (4.8)	25 (2.9)
**Urbanicity**				
Large city	254 (27.2)	233 (25.0)	96 (14.3)	150 (17.2)
Suburbs of a large city	331 (35.4)	177 (19.0)	264 (39.5)	178 (20.4)
Small city	185 (19.8)	107 (11.5)	154 (23.0)	98 (11.2)
Town or village or rural area	164 (17.6)	415 (44.5)	155 (23.2)	446 (51.1)
**Essential worker in family**				
No	638 (68.8)	631 (69.1)	378 (56.8)	468 (56.2)
Yes	230 (24.8)	224 (24.5)	233 (35.0)	289 (34.7)
Yes, COVID facility	59 (6.4)	58 (6.4)	54 (8.1)	76 (9.1)
**Any family impact**				
No	539 (58.5)	551 (59.4)	491 (73.4)	574 (66.4)
Yes	383 (41.5)	377 (40.6)	178 (26.6)	291 (33.6)
**Family member diagnosed**				
No	877 (94.5)	876 (93.6)	651 (96.9)	829 (94.5)
Yes	51 (5.5)	60 (6.4)	21 (3.1)	48 (5.5)
**2-Week exposure**				
None	818 (87.9)	789 (84.1)	643 (95.7)	782 (89.4)
Exposure to person with symptoms	77 (8.3)	100 (10.7)	17 (2.5)	68 (7.8)
Exposure to person with diagnosis	36 (3.9)	49 (5.2)	12 (1.8)	25 (2.9)
**2-Week symptom count**				
None	588 (62.9)	539 (57.5)	566 (84.1)	684 (78.0)
One	196 (21.0)	201 (21.4)	77 (11.4)	119 (13.6)
Two	72 (7.7)	85 (9.1)	17 (2.5)	44 (5.0)
Three or more	79 (8.4)	113 (12.0)	13 (1.9)	30 (3.4)

Because all data were collected anonymously, no IRB oversight was required. Exemption from IRB oversight was approved by the Advarra Institutional Review Board. Participants using the PA website are required to agree to the Terms of Service notification (https://prolific.ac/assets/docs/Participant_Terms.pdf) before being allowed to complete surveys. Per the IRB exemption, no additional informed consent was required.

### Measurement domains

To assess the structure, psychometric properties, and construct validity of the CRISIS, we focused on the following domains and indicators (see Supplement for more details; Supplemental Fig. 1 for the list of items, and Supplemental Information for the full written CRISIS questionnaire):

#### Participant characteristics

Age, sex, race/ethnicity, self- or parent-rated health, urbanicity, education, household size, health insurance coverage, and family’s receipt of government assistance. Race was reported to Prolific Academic and combined with a question on Hispanic ethnicity to generate the following categories: Hispanic, non-Hispanic white, non-Hispanic black, Asian, and other.

#### SARS-CoV-2 exposure/infection in the past 2 weeks

Possible exposure to SARS-CoV-2, possible symptoms of COVID-19, family member diagnosis of COVID-19, essential worker in the household, and whether there had been any impacts on family members such as hospitalization, quarantine, and job loss because of COVID-19.

#### COVID worries in the past 2 weeks

Participants reported on a five-point Likert scale how worried they have been during the past 2 weeks about infection, friends and family being infected, and possible impacts on physical and mental health, as well as time spent reading or talking about COVID-19, and hope that the pandemic will end soon.

#### Life changes due to the pandemic in the past 2 weeks

Downstream and subjective impacts of structural changes, such as changes in social contacts, effects on family relationships, changes in living situation, food insecurity, and stressors associated with these changes (14 items). Participants were also asked about job loss and school closure due to the pandemic; these items were used as internal validators.

#### Mood states

Ten items from the circumplex model of affect^30,31^ were included to measure mood/anxiety, both during the past two weeks (hereafter referred to as “Current Mood States”) and during the three months prior to the pandemic (hereafter referred to as “Prior Mood States”).

#### Substance use

Frequency of use of tobacco, alcohol, marijuana, and other substances during the past two weeks and during the three months prior to the pandemic.

#### Daily behaviors

Average weekday and weekend bedtime and sleep duration, frequency of exercise, time spent outdoors, and length of media use per day were rated for the past two weeks and the three months prior to the pandemic.

### Analysis

#### Overview

Analyses focused on five domains of interest: COVID Worries, Life Changes, Mood States, Substance Use, and Daily Behaviors. The statistical approaches are described below, with additional details in the Supplement. Structure was assessed via factor analysis and community detection subtyping. Test–retest reliability was measured via the Intraclass Correlation Coefficient (ICC(3,1))^33^. Construct validity was assessed by comparing associations between domains using chi-squared tests, ANOVAs, ANCOVAs, and via random forests.

#### Factor analysis (Aim 2)

Confirmatory factor analysis (CFA) was performed in each sample. To assess the stability and reproducibility of our factor structure, we split the 2/3 training dataset further into two datasets each corresponding to 1/3 of the full dataset, and we performed CFA on each. To assess unidimensionality, CFA was applied in each sample split, with a comparative fit index (CFI) of > 0.95 and an Omega of > 0.8 indicating adequate fit^34^. Resulting factor scores were used to assess construct validity for Aim 4. To explore the dimensionality of these domains to assist in future studies, we also conducted an exploratory factor analysis of each of our domains in these samples as well and have included these results in the Supplement.

#### Community detection based subtyping (Aim 2)

Louvain community detection (LCD)^35^ was used to derive data-driven subtypes on domains that exhibited poor unidimensional fit in CFA. In order to maximize the modularity of the sample, LCD selects the cluster resolution that maximizes the within-community coherence and between-community segregation. LCD^35^ was enhanced through bootstrap aggregation (i.e., bagging) which has been shown to generate more reproducible clusters (see Supplemental Methods)^36,37^. Resulting subtypes were used to assess construct validity for Aim 4.

#### Test–retest reliability (Aim 3)

We assessed the reliability of the factor scores and individual items using intraclass correlation coefficient (ICC 3,1)^33^ on the separate 24 h test retest sample for each of the US and UK adult and parent report samples. ICC results for individual items on Daily Behaviors, Substance Use, and Life Changes are summarized in the Supplemental Table 2.

#### Random forests (Aim 4)

Random forest (RF), a robust technique known for its ability to model dependencies between predictor variables (See Supplemental Methods)^38,39^, was used to examine associations of participant characteristics with Mood States and Behaviors (e.g., COVID Worries, Life Changes, Daily Behaviors, Media Use, Substance Use, and Prior Mood States). RF assesses performance across the ensemble of decision trees on the samples not included in each bootstrap iteration. The out-of-bag mean square error (MSE) and node impurity were used as measures of relative variable importance for each predictor. Generalizability of the performance and importance of the variables identified in each random forest analysis was assessed in the 1/3 hold-out datasets. In each subsample, individual variable correlations and linear regression models were trained using the four most important variables and their interaction terms. These models were then applied to each corresponding hold-out set to evaluate out-of-sample performance.

## Results

### Acceptability and feasibility (Aim 1)

Characteristics of the total analytic sample are presented in Table 1. Supplemental Table 1 shows the amount of missing data and time to completion. The proportion of complete surveys was high (95.2%). The numbers of missing items per survey were low, on average 0.6 (SD = 1.8) and 0.5 (SD = 1.1) for the US and UK Adult reports respectively, and 0.4 (SD = 0.9) and 0.4 (SD = 1.0) for the US and UK parent reports. There was an average of 13.9 (SD = 13.1) and 14.4 (SD = 11.5) minutes to completion for the US and UK Adult reports respectively and 14.1 (SD = 7.5) and 13.9 (SD = 20.9) for the US and UK parent reports. Feedback from the open-ended questions was generally positive, and no comments suggested that CRISIS was a burden, consistent with the high completion rate and low rate of missing values.

### Factor analysis and community detection subtyping (Aim 2)

To evaluate the structure of the five domains of interest, we first conducted confirmatory factor analysis (Table 2). The Mood States and COVID Worries domains each demonstrated high internal consistency as assessed using coefficient Omega (> 0.8), and good unidimensional model fit as assessed using the comparative fit index (CFI > 0.95) across each split samples of US and UK in both the Adult Self-Report and Parent-Report data. Associations between factor scores are depicted in Supplementary Fig. 2. We did not find strong evidence for unidimensional model fit for Daily Behaviors and Media Use (CFI < 0.9), Life Changes (Adult CFI < 0.9; Parent CFI < 0.95), or adult Substance Use (CFI > 0.95 in US and < 0.75 in UK), which was expected given that these domains were designed to capture a broad variety of behaviors. We have included both EFA results of these domains on the first half sample as well as the complete CFA results in the supplemental information.

**Table 2:** Fit statistics from confirmatory factor analysis in split-half samples from adult self-report and parent respondents in the US and UK.

	Sample 1 CFI fit US | UK	Sample 2 CFI fit US | UK	Sample 1 omega (ω) US | UK	Sample 2 omega (ω) US | UK
**Adult report**				
COVID worries	0.99 | 0.99	0.99 | 0.99	0.88 | 0.81	0.88 | 0.84
Life changes	0.82 | 0.86	0.87 | 0.87	0.76 | 0.76	0.79 | 0.79
Mood States	0.98 | 0.99	0.99 | 0.99	0.90 | 0.92	0.91 | 0.91
Substance Use	0.98 | 0.98	0.97 | 0.98	0.73 | 0.68	0.71 | 0.60
Daily behaviors	0.88 | 0.88	0.89 | 0.88	0.63 | 0.71	0.76 | 0.73
**Parent report**				
COVID worries	1.00 | 0.99	0.99 | 0.99	0.90 | 0.86	0.89 | 0.85
Life changes	0.94 | 0.92	0.94 | 0.94	0.77 | 0.75	0.77 | 0.73
Mood states	0.99 | 0.98	0.97 | 0.98	0.91 | 0.87	0.89 | 0.89
Daily behaviors	0.77 | 0.93	0.88 | 0.95	0.80 | 0.87	0.84 | 0.86

**Figure 1:**
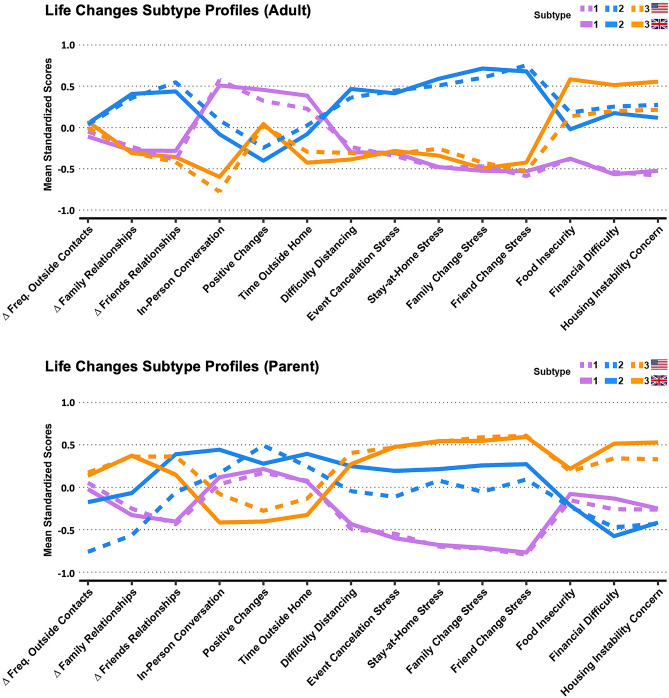
Life Changes Subtype profiles from adult self-reports and parent reports. Mean normalized profile loadings are displayed on the y-axis. US subtypes in solid lines, UK in dashed lines. Adult Subtypes: Purple (1): *low stress*, Blue (2): *social/interpersonal stress*, Orange (3): *economic stress.* Parent-Report Subtypes: Purple (1): *low stress*, Blue (2): *social stress,* Orange (3): *social/economic stress* Notes: ∆ Family Relationships and ∆ Friends Relationships are coded so that higher scores indicate worsening quality of relationships. Prior to the community detection analyses In-Person Conversation was re-coded into tertiles.

Because the Life Changes, Substance Use, and Behavior & Media Use domains generally exhibited poor unidimensional fit in CFA, they were summarized via community detection subtyping. We conducted two subtyping analyses: one focused on the Life Changes domain, and another focused on the combined Substance Use and Behavior and Media Use domains, using questions pertaining to the 3 months prior to the pandemic (referred to as Prior Habits). The derived Life Changes subtypes in adults and children are displayed in Fig. 1. For both the adult self-report and parent report, the Life Changes subtypes were highly reproducible across the US and UK samples, as Pearson’s correlations across the US and UK profiles show high consistency (r = 0.91–0.99). Prior Habits subtypes in adults and children are described in the supplemental results.

In the adult sample, the *low stress* subtype (purple; 1) reported greater positive changes, in-person conversations, and time outside; lower levels of stress from distancing, cancellations, and relationship changes; and lower levels of food insecurity, financial difficulty, and housing instability. The *social/interpersonal stress* subtype (blue; 2) reported worsening of relationships, higher stress levels, few positive changes, and moderate levels of economic concerns. The *economic stress* subtype (orange; 3) reported the most problems with food security, financial difficulty, and housing stability, but lower levels of other stresses, while also having the least in-person conversations.

In the parent report, the *low stress* subtype (purple; 1) had somewhat improved family and friend relationships, low stress related to social and interpersonal changes, and average levels of economic stress, time outside, and positive changes. The *social stress* subtype (blue; 2) reported moderate to high levels of stress related to social and interpersonal changes; low levels of economic stress; higher levels of positive changes, time outside, and in-person conversations; US parents reported more worsening of relationships than did UK parents. The *social/economic stress* subtype (orange; 3) reported the highest levels of economic and social/interpersonal stress, worsening of family relationships, and the lowest levels of positive changes, time outside the home, and in-person conversations.

### Test–retest reliability (Aim 3)

We found the Mood States and COVID Worries factor 24 h reliabilities were high (ICC (3,1) = 0.79–0.87) in all Adult and Parent Report samples. Reliability of single items not included in factor scores was generally moderate to excellent (ICC of Prior Habits variables mean = 0.79, sd = 0.09; ICC of Life Changes variables mean = 0.64, sd = 0.15; ICC of Substance Use variables mean = 0.88, sd = 0.19) and are presented in Supplemental Table 2.

### Associations between domains (Aim 4)

Mean factor scores by participant characteristics among adults are presented in Table 3 and parent reports are shown in Supplemental Table 3. Most associations were consistent across US and UK adults. COVID Worries was consistently higher among those with any family impact (US *p* < 0.001; UK *p* < 0.001), a family member with a COVID diagnosis (US *p* < 0.05; UK *p* < 0.005), and potential symptoms (US *p* < 0.001; UK *p* < 0.001). In addition to associations with age and sex, Mood States scores were also consistently higher among those with any family impact (US *p* < 0.05; UK *p* < 0.005), exposure to someone with symptoms (US *p* < 0.05; UK *p* < 0.05), and potential symptoms (US *p* < 0.001; UK *p* < 0.001). COVID Worries and Mood States factor scores were not associated with participant race/ethnicity or having an essential worker in the home.

**Table 3:** Mean factor scores for unidimensional constructs among adults by demographic and COVID-related characteristics.

	Adult US			Adult UK		
	Mean (SD)			Mean (SD)		
	Covid worries	Prior mood states	Current mood states	Covid worries	Prior mood states	Current mood states
**Population**	0.003 (0.85)	0.0001 (0.63)	0.0006 (0.73)	0.0002 (0.82)	0.006 (0.66)	0.006 (0.73)
**Sex**		*	***	***	**	***
Male	− 0.02 (0.83)	− 0.06 (0.63)	− 0.09 (0.70)	− 0.18 (0.83)	− 0.08 (0.67)	− 0.17 (0.72)
Female	0.02 (0.97)	0.04 (0.64)	0.06 (0.75)	0.14 (0.79)	0.08 (0.64)	0.14 (0.70)
**Age**	***	***	***		***	***
Under 30	− 0.23 (0.83)	0.10 (0.63)	0.11 (0.71)	− 0.05 (0.82)	0.18 (0.62)	0.20 (0.72)
30–49	0.16 (0.85)	0.02 (0.62)	0.10 (0.74)	0.07 (0.80)	0.08 (0.63)	0.07 (0.68)
50 and older	− 0.23 (0.83)	− 0.22 (0.60)	− 0.34 (0.67)	− 0.05 (0.86)	− 0.32 (0.62)	− 0.31 (0.72)
**Race**						
Asian	0.0 (0.82)	0.06 (0.59)	− 0.06 (0.64)	0.05 (0.90)	0.01 (0.59)	0.12 (0.73)
Black	− 0.07 (0.99)	0.03 (0.70)	− 0.01 (0.82)	− 0.08 (0.87)	− 0.13 (0.58)	− 0.11 (0.71)
Hispanic	0.17 (0.84)	− 0.01 (0.66)	0.08 (0.81)	0.10 (0.96)	0.3 (0.67)	0.42 (0.85)
Other	− 0.05 (0.82)	0.08 (0.62)	0.15 (0.71)	0.04 (0.73)	0.04 (0.67)	− 0.001 (0.75)
White	0.0 3(0.84)	− 0.02 (0.63)	− 0.02 (0.72)	− 0.001 (0.02)	0.01 (0.66)	− 0.01 (0.72)
**School closed**	**	**	***		*	***
School closed but classed resumed online	0.15 (0.86)	0.13 (0.59)	0.13 (0.70)	0.01 (0.81)	0.18 (0.64)	0.29 (0.72)
School closed but classes did not resume	− 0.56 (0.93)	0.25 (0.80)	0.20 (0.96)	0.09 (0.98)	0.02 (0.55)	0.29 (0.70)
School Did Not Close	0.09 (1.12)	0.16 (0.70)	0.12 (0.61)	0.15 (0.94)	0.09 (0.53)	− 0.02 (0.90)
Not APPLICABLE	− 0.03 (0.83)	− 0.04 (0.64)	− 0.04 (0.73)	− 0.01 (0.81)	− 0.02 (0.66)	− 0.04 (0.72)
**Job loss**	*	*				
Job prior to pandemic and still working	− 0.01 (0.84)	− 0.06 (0.64)	− 0.02 (0.73)	0.01 (0.83)	− 0.01 (0.62)	0.06 (0.67)
Job prior to pandemic and not still working	0.12 (0.84)	0.07 (0.61)	0.12 (0.72)	0.001 (0.82)	0.07 (0.59)	− 0.01 (0.71)
Did not have job prior to pandemic	− 0.09 (0.90)	0.05 (0.64)	− 0.07 (0.75)	0.01 (0.83)	− 0.03 (0.75)	− 0.04 (0.72)
**Essential worker in family**						
No	− 0.01 (0.84)	− 0.01 (0.62)	− 0.02 (0.73)	− 0.04 (0.81)	− 0.002 (0.65)	− 0.02 (0.72)
Yes	− 0.003 (0.91)	0.02 (0.69)	0.05 (0.75)	0.04 (0.85)	− 0.02 (0.66)	0.03 (0.72)
Yes, works in COVID facility	0.17 (0.80)	0.05 (0.55)	0.05 (0.68)	0.13 (0.89)	0.04 (0.53)	0.11 (0.66)
**Any family impact**	**	*	***	***	**	***
No	− 0.07 (0.88)	− 0.04 (0.64)	− 0.08 (0.71)	− 0.09 (0.94)	− 0.04 (0.66)	− 0.11 (0.70)
Yes	0.11 (0.79)	0.07 (0.62)	0.13 (0.74)	0.14 (0.78)	0.08 (0.64)	0.18 (0.74)
**Family member diagnosed**	*		*	**		
No	− 0.01 (0.81)	− 0.01 (0.64)	− 0.02 (0.72)	− 0.02 (0.82)	0.01 (0.65)	− 0.01 (0.72)
Yes	0.28 (0.80)	0.08 (0.63)	0.25 (0.87)	0.30 (0.83)	− 0.02 (0.69)	0.18 (0.80)
**2-Week COVID exposure**		*	**	**	*	**
None	− 0.02 (0.86)	− 0.01 (0.64)	− 0.02 (0.73)	− 0.03 (0.82)	− 0.02 (0.66)	− 0.03 (0.72)
Exposure to person with symptoms	0.15 (0.74)	0.18 (0.59)	0.26 (0.73)	0.10 (0.81)	0.15 (0.58)	0.21 (0.75)
Exposure to person with diagnosis	0.20 (0.85)	− 0.05 (0.62)	− 0.02 (0.75)	0.37 (0.81)	0.08 (0.69)	0.21 (0.75)
**2-Week symptom count**	***	***	***	***	***	***
None	− 0.09 (0.84)	− 0.08 (0.61)	− 0.10 (0.72)	− 0.13 (0.80)	− 0.10 (0.67)	− 0.14 (0.72)
One	0.16 (0.89)	0.17 (0.67)	0.18 (0.73)	0.17 (0.86)	0.14 (0.60)	0.20 (0.66)
Two	0.21 (0.78)	0.05 (0.65)	0.11 (0.71)	0.22 (0.84)	0.21 (0.64)	0.26 (0.73)
Three or more	0.13 (0.84)	0.17 (0.60)	0.22 (0.71)	0.20 (0.74)	0.15 (0.61)	0.16 (0.73)

Significant group differences are represented by asterisks, uncorrected for multiple comparisons: *p < 0.05, **p < 0.01, ***p < 0.001.

**Figure 2:**
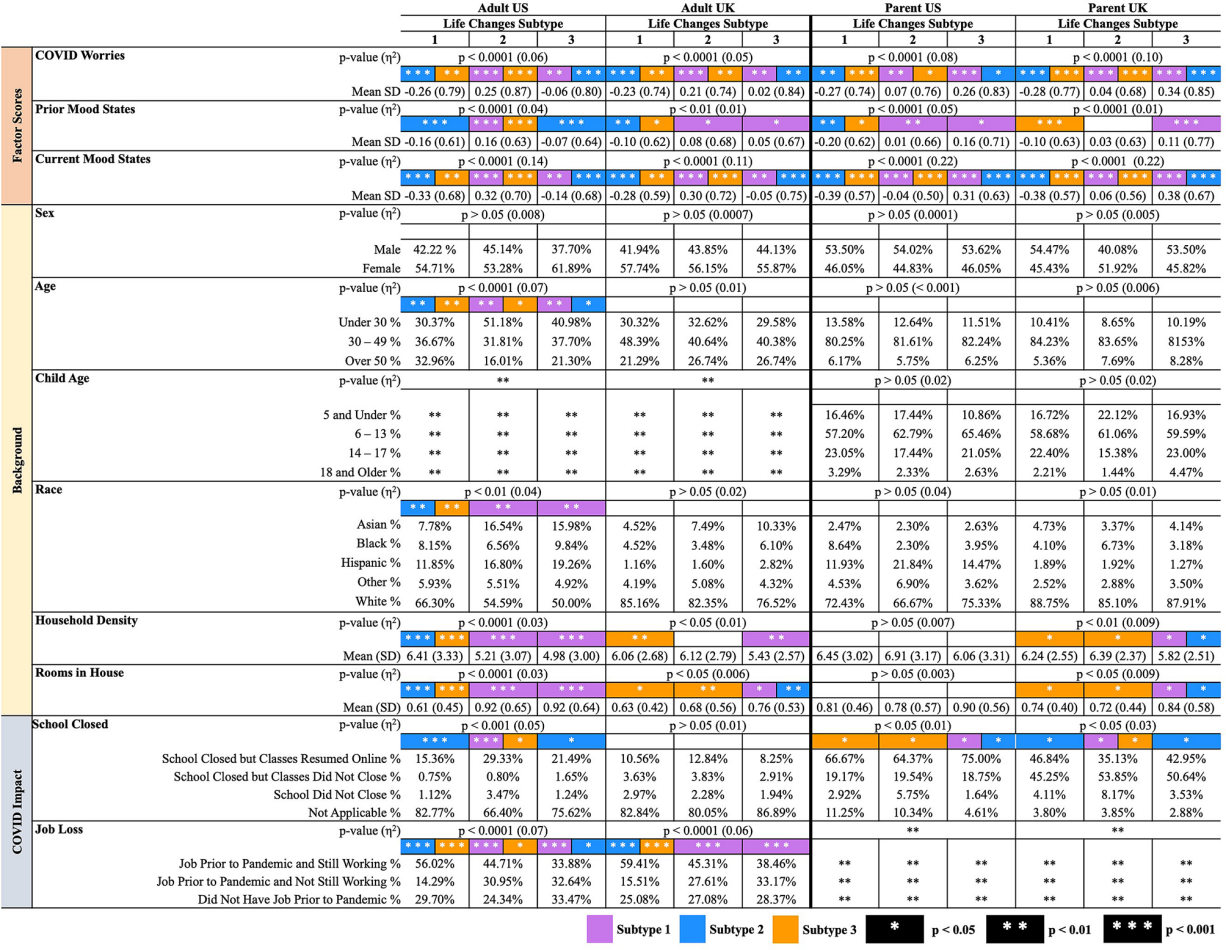
Mean factor scores, demographic characteristics, and pandemic-related school closure and job loss by Life Changes subtypes. Color indicates that the group in a given column is significantly different from the subtype of the indicated color. Pairwise group differences are represented by white asterisks: *p < 0.05, **p < 0.01, ***p < 0.001.

Associations of Life Changes subtypes with factor scores, participant characteristics, and school closure and job loss are presented in Fig. 2. Life changes subtype was associated with Mood States (Prior: US *p* < 0.00001, UK *p* < 0.00001; Current: US *p* < 0.00001, UK *p* < 0.00001), as well as COVID Worries score (US *p* < 0.00001, UK *p* < 0.00001). Adjusting for Prior Mood States, Current Mood States scores were highest in the *social/interpersonal stress* subtype among adults and the *social/economic stress* subtype among parent reports (Fig. 2). Life Changes subtypes also differed by key demographic characteristics including age, race/ethnicity, education, rooms in house, household density, and employment. Corresponding results for Prior Habits subtypes appear in Supplemental Fig. 3 and Fig. 4. Briefly, we find differences between Prior Habit subtypes in COVID Worries, Prior Mood States, and Current Mood States, indicating the importance of prior behavioral and psychological states in influencing the negative mental health outcomes of the pandemic. Chi-square tests of Life Changes subtypes also show significantly different proportions across Prior Habit subtypes (Supplemental Table 4).

### Predicting mood states in the early phase of the pandemic (Aim 4)

**Figure 3:**
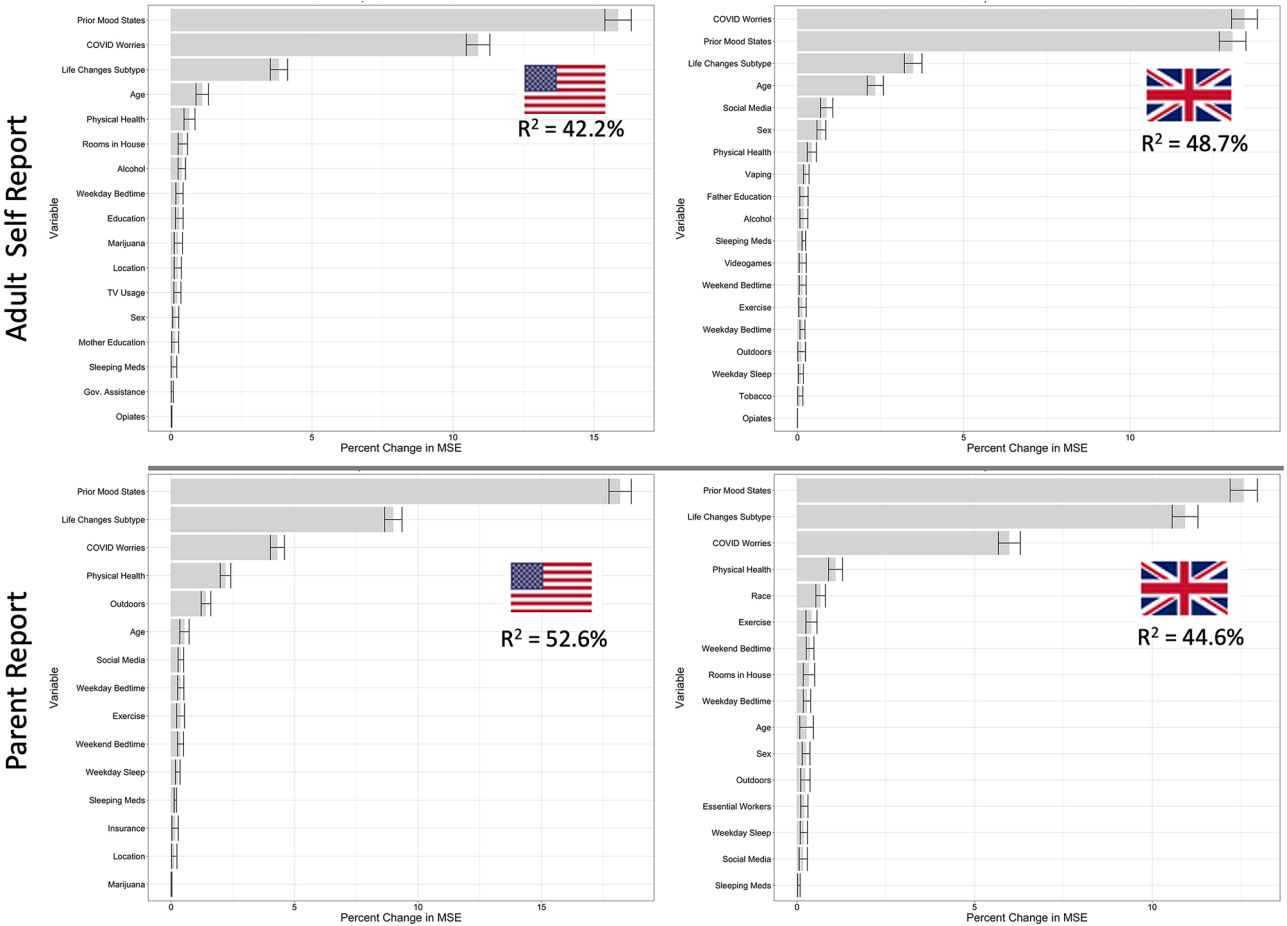
Variable importance and overall performance of Random Forest models predicting Current Mood States in the US and UK in both Adult self-report and parent report data. Variables are ranked by importance as measured by out-of-bag change in mean squared error (MSE), and those with a 95% lower bound above zero are shown here. Variables included: Prior Mood States, Life Changes Subtype, COVID Worries, physical health, age, sex, outdoors, exercise, social media, TV, videogame, weekend bedtime, weekend sleep, weekday bedtime, weekday sleep, insurance, rooms in house, government assistance, number in household, essential worker in household, Marijuana, Alcohol, vaping, opiates, sleeping medication and other drug use.

The random forests estimation of the relative importance of demographics, Prior Mood States, COVID Worries, Life Changes subtype, and Prior Behavior, Media, and Substance Use, in predicting Current Mood States are shown in Fig. 3. Variables are ranked along the y-axis according to their importance in predicting out-of-bag Current Mood States as measured by the percent change in MSE, displayed on the x-axis. Models accounted for a high percentage of the out-of-bag variance in Current Mood States across all samples (Adult; US: 42.2%, UK: 48.7%; Parent; US: 52.6%, UK: 44.6%).

Prior Mood States, COVID Worries, Life Changes Subtype, and age were the most important domains for predicting Current Mood States in descending order of importance in the adult sample. On the other hand in the parent report sample, after prior Mood states, the Life Changes subtype, COVID Worries and parent-rated health predicted Current Mood States. Across the US and UK these importance values were highly replicable (Adult Self Report: MSE Pearson’s r = 0.98; Impurity Pearson’s r = 0.99. Parent Report: MSE Pearson’s r = 0.96; Impurity Pearson’s r = 0.98).

To assess the successive impact of adding COVID Worries and the Life Changes Subtype to our predictions, we tested a baseline model without these variables and found much lower predictive accuracy (Adult; US: 30.4%, UK: 28.0%; Parent; US: 35.8%, UK: 20.7%). Performance increased dramatically when adding either COVID Worries (Adult; US: + 10.0%, UK: + 16.8%; Parent; US: + 7.9%, UK: + 12.4%) or Lifestyle Changes Subtype (Adult; US: + 5.5%, UK: + 7.2%; Parent; US: + 13.3%, UK: + 17.4%). As expected based on the performance and variable importance ranking of the full models, COVID Worries conferred more additional predictive performance for the Adult sample, while Lifestyle Changes Subtype conferred more additional predictive performance for the Parent report sample.

Based on the random forest results, the four most important predictors of Current Mood States in adults were Prior Mood States, COVID Worries, Life Changes Subtype, and age; in children, next most important was a variable of parent-rated health of the child. Our trained linear model, including interactions, was able to predict between 49.6 and 56.5% of the variance in Current Mood States across all four of the 1/3 sample hold-out data (Supplemental Table 5). This result strongly demonstrates the generalizability of the importance of these variables in predicting Current Mood States. We tested whether these variables would show similar strength of association with Current Mood States in the hold out sample as well and found each to be very similar as in the training sample (Supplemental Table 6).

## Discussion

Our results support the theoretical framing from work on the public health implications of disasters such as COVID-19, namely that perceived risk of COVID-19, prior mental health status, and lifestyle changes would be key predictors of current mood states during the pandemic^23,28,40,41^. These results support prior work suggesting that individual differences in the stresses associated with changes in lifestyle are one of the most important correlates of the pandemic’s effect on mood and anxiety^42,43^. Future research and intervention efforts may benefit from focusing on how to ameliorate the negative ramifications of social isolation and economic insecurity that we find related with the worst mental health outcomes in children and adults, in particular those already with a history of mental health problems.

The findings presented here demonstrate the feasibility, reliability, and construct validity of the CRISIS in large pilot samples in the US and UK. The high completion rates, low rates of missing data, and rapid completion times demonstrate that the CRISIS is feasible to administer in large samples. The unidimensional Mood States and COVID Worries factor scores reached excellent levels of both internal and test retest reliability (Omega > 0.9; ICCs between 0.79 and 0.87), and individual items from other measured domains showed high ICC as well. High reliability of survey instruments is absolutely critical in evaluating change and in robustly identifying those in need of interventions or other forms of support. The unidimensional structure of the COVID Worries and Mood States domains were highly replicable across all samples. The construct validity of the CRISIS was demonstrated by the reproducible associations between measured domains as well as the associations of COVID Worries and pandemic-associated life changes with Current Mood States determined from the well-established circumplex model of affect^30,31^. Together, the results demonstrate the utility of the CRISIS for population-based mental health research during the COVID-19 pandemic. The highly robust replication of our findings across samples, countries, and informants suggests that CRISIS would be appropriate for application across an array of research settings around the world. To date, the CRISIS is being administered in more than eight countries, and translations have been developed in several languages. Thus, we will have the opportunity to test the reliability and validity in middle- and low-income countries around the globe. More about CRISIS and its adaptation for Autism and related Neurodevelopmental Conditions (CRISIS AFAR; www.crisissurvey.org/), and other such international collaborations through the Wellcome Trust (www.COVIDminds.org/) can be found online.

COVID Worries was either the first (UK) or second (US) most important predictor of Current Mood States among adults in April, 2020, followed by pandemic-associated life changes. These results suggest that fear and worry about COVID and resulting changes in routines and daily life are significant drivers of adverse mental health outcomes associated with the pandemic, consistent with established perspectives on COVID-19^44–46^ and previous data on the impact of the Fukushima disaster^47,48^. This speaks to the value of measuring COVID-related fears and worries, as in the CRISIS and other instruments developed for the COVID pandemic^21–27,49,50^.

Our finding of parent report data that indicated that Current Mood States among children was more strongly related to Life Changes than COVID Worries is consistent with the known importance of regular, predictable, daily routines for pediatric mental health^52–54^, and suggests that attending to changes in children’s lives may be key to predicting those at greatest risk for negative psychological impact of the pandemic. Consistent with the review of Brooks et al.^9^, subgroups reporting family and social isolation stress in both adults and children in the US and UK had significantly higher Current Mood States scores. In addition, subgroups of children with higher family and social isolation stress also experienced the highest parent-reported stress related to financial and food security. This underscores the impact of multifactorial physical, emotional, interpersonal, social, and financial stressors that converge during this pandemic. The links between Life Changes profiles and the COVID Worries and Mood States factors attest to the validity of these domains and their potential utility as targets for pandemic-related interventions. It also implies that active steps that could be taken to offset the impact and lessen the burden of changes in lifestyle by social, governmental or other agencies could have a significant impact in ameliorating negative mental health outcomes^51^. Future studies including repeated longitudinal assessments could assess the potential long-term effects of such policies on mental health and enable comprehensive evaluation of costs and benefits.

Possessing information on mental and behavioral health prior to the pandemic significantly enhances the ability to assess the impact of the pandemic and its correlates on mental health outcomes by allowing researchers to evaluate both the change in mental health during the pandemic and the potential for prior characteristics to moderate the effects of pandemic-related stressors. Indeed, this is a central goal of the CRISIS initiative. Because information on prior mental health may not be available to all researchers, we included retrospective reports of key domains in the CRISIS to enable researchers to evaluate the role of prior characteristics and clinical state^55^. Our finding that Prior Mood States and Prior Habits were significantly associated with Current Mood States provides support for the importance of psychological status prior to the pandemic. The reproducibility of the structure of the multi-dimensional domains of Life Changes and Prior Habits attests to the value of this feature of the CRISIS. Ultimately, prospective measures of pre-COVID mental health will facilitate the identification of those at greatest risk of long term sequelae of this pandemic, as shown in previous disaster research^2,6^.

This study is limited by its use of a web-based convenience sample, which raises the possibility of selection bias and impedes our ability to generalize our findings to the broader US and UK populations. This limitation applies to the majority of the current mental health surveys of COVID-19, which have mostly used samples ascertained through web-based sources^56^. We employed this approach in order to quickly deploy the CRISIS to large numbers of participants within a brief time frame during the initial peak of the pandemic, to rapidly evaluate test–retest reliability, and to pilot the shorter follow-up version of the CRISIS. We do not expect the composition of our sample to strongly affect our findings regarding the structure of the CRISIS, the reliability of its items or unidimensional domains, or its construct validity. The relatively lower racial/ethnic diversity in the present study is an important limitation, particularly in light of the disproportionate impact of COVID-19 on marginalized communities^57,58^. Further work is required to assess the properties of CRISIS in different racial/ethnic groups, cultural settings, and languages. However, these data sources provided samples with broad coverage of the US and UK populations with respect to age, sex, and race^57,59,60^. Moreover, this pilot study did not include the youth self-report version of the CRISIS, but this work is now underway in our collaborative network. Validation of youth reports will be particularly relevant because the profiles of predictors of change derived from families with children under age 18 differed from those from adult households.

These findings reflect the initial steps of instrument development to implement our collaborative effort on the mental health impact of the COVID-19 pandemic. With some exceptions^21–27^, this study of the CRISIS is one of the few COVID-19 stress/anxiety questionnaires that has provided psychometric data on its factor structure and/or validity of the content. The factors derived in our study replicate those of Taylor et al.^24^ who likewise employed statistical approaches that demonstrated the heterogeneity of impact of COVID-19 fears and anxiety, and the impact of prior mental health problems on its severity. We further demonstrate the utility of the CRISIS through its reproducible structure, its acceptability, feasibility, and reliability, and its construct validity across multiple samples. The inclusion of adult and parent versions to examine differences in the impact of COVID-19 across the life span will also facilitate our ability to gain insight into the impact of the pandemic on children and families. Efforts to administer the survey in previously well characterized samples such as the Healthy Brain Network, a landmark ongoing mental health study of 10,000 children with deep phenotyping across a range of psychiatric, cognitive, affective, language, genetic, and neuroscientific characteristics^61^, are underway. The major goal of our initiative is to conduct research that informs priorities for interventions and policy changes to ameliorate the mental health consequences of the pandemic, both acutely and in the long term.

## Supplementary Information

Supplementary Information.
