# Supplementary material for: The Coronavirus Health and Impact Survey (CRISIS) reveals reproducible correlates of pandemic-related mood states across the Atlantic
Source: Sci Rep. 2021 Apr 14;11:8139. doi: 10.1038/s41598-021-87270-3 (PMC8046981; doi:10.1038/s41598-021-87270-3)
Supplement: Supplementary file 1 — Supplementary Information. [file 41598_2021_87270_MOESM1_ESM.pdf]

Supplemental Materials

The Coronavirus Health and Impact Survey (CRISIS) reveals reproducible correlates of pandemic-related mood states across the Atlantic.

Aki Nikolaidis[1], Diana Paksarian[2], Lindsay Alexander[1], Jacob Derosa[1], Julia Dunn[2], Dylan M. Nielson[3], Irene Droney[1], Minji Kang[1], Ioanna Douka[3], Evelyn Bromet[4], Michael Milham[1,5], Argyris Stringaris[3], Kathleen R. Merikangas[2,6]

1 – Center for the Developing Brain, The Child Mind Institute, New York, NY.
2 – Genetic Epidemiology Research Branch, Intramural Research Program, National Institute of Mental Health, Bethesda, MD
3 – Section on Clinical and Computational Psychiatry (CompΨ), National Institute of Mental Health, National Institutes of Health, Bethesda, Maryland
4 – Department of Psychiatry, Renaissance School of Medicine at Stony Brook University.
5 – Nathan Kline Institute for Psychiatric Research
6 – Johns Hopkins Bloomberg School of Public Health

Corresponding Author: Aki Nikolaidis; g.aki.nikolaidis@gmail.com

Supplemental Methods

Overview: First, we used confirmatory factor analysis to assess the structure of each of the domains and inform the calculation of factor scores for unidimensional constructs. Test-retest reliability was calculated for unidimensional factors and individual items using ICC(3,1).[33] We used Louvain community detection, a clustering technique, to meaningfully summarize domains for which unidimensional factors exhibited poor fit. Finally, we used random forests to demonstrate the construct validity of the CRISIS by assessing the importance of the included domains in predicting the Current Mood States factor.

Subtyping: We use bagging-enhanced Louvain Community Detection to discover groups of individuals that have profiles across both the Life Changes questions, and the Daily Behaviors, Media Use, and Substance Use questions, which we called the Prior Habits subtypes. Louvain Community Detection is known to robustly link observations together through the use of an iterative modularity-optimizing procedure to find groups of individuals. Other clustering approaches, such as K-means, or spectral clustering, require the experimenter to choose the resolution of the clustering a priori, which can be problematic and lead to instability across samples.[63–65] Louvain Community detection on the other hand, through iterative permutations of individuals in each community, optimizes for modularity, a commonly used metric of cluster quality.[35,66] We enhance the reproducibility of our subtyping method through the use of bootstrap aggregation, or bagging. Using bootstrap aggregated clustering creates more reproducible clusters by reducing variability that may occur due to random variations in sample composition.

Random Forest: Briefly, the RF algorithm creates a series of decision trees for which a random selection of variables are chosen and a bootstrapped sample is used to train the model. For each iteration of the 1000 bootstrap runs, the performance on each of these decision trees on the out-of-sample data, roughly ⅓ of the sample, is aggregated and used to assess the performance of the RF model. (For a review of RF, see[65]). RF provides a robust assessment of the relative impact of each of these variables in predicting outcomes, known as variable importance, which we assess for each variable in our predictive model. To protect against overfitting, we create a null performance distribution from our own data by shuffling the outcome variable and repeating the random forest prediction pipeline 1000 times. The out-of-sample prediction R-squared value is then calculated for our prediction and compared to the distribution of these 1000 shuffled null models. We assess if our predictive accuracy surpasses the 99.9999% confidence interval of the null model. We used COVID Worries and Prior Mood States transformed into quintiles in order to protect against the inflation effects that RF can have on the variable importance of continuous versus categorical variables.[67]

Supplemental Results

Prior Habits Subtyping: We found three Prior Habits subtypes in both US and UK adult samples. Individuals in subtype one spend the least amount of time exercising and outdoors, and in the US go to sleep relatively later and spend more time on media, while in the UK this subtype goes to sleep earlier and spends an average amount of time on media. Subtype two goes to bed later, in the US this subtype gets relatively less sleep but spends more time exercising and outdoors, while in the UK this group gets an average amount of sleep and time spent exercising and outdoors. Subtype three goes to bed earliest and spends more time exercising and outdoors, and reports relatively lower media use and drug use.

Across US and UK samples these subtypes were highly reproducible, with high Pearson's correlations between subtype mean scores ($r = 0.71 – 0.96$) for the adult sample. ANOVA revealed Prior Habits subtypes had different Prior Mood States factor scores (US $p < 0.00001$, UK $p < 0.00001$) but ANCOVA of Current Mood States controlling for Prior Mood states only differed in the US sample (US $p < 0.05$, UK $p > 0.05$). The US sample also showed different Mood States scores by subtype (US $p < 0.00001$; UK $p = 0.06$). COVID Worries scores differed by subtype in the UK but not US (US $p > 0.05$, UK $p < 0.01$). Mean factor scores by subtype are shown in the Supplemental table 3. Overall, results indicated significant differences between subtypes in prior mood states scores in both US and UK, with subtype one showing highest scores in the US and subtype 3 showing highest scores in the UK. We also see significant subtype differences by age in both US and UK. Prior Habits subtypes also differed by key demographic variables including age, race, education, rooms in house, household density, and employment status (see Supplement).

The parent report US and UK subtypes were highly reproducible, with high Pearson's correlations between subtype mean scores ($r = 0.97 – 0.99$) In the parent report data we found three Prior Habit subtypes in the US sample and four in the UK sample. Individuals in subtype one, in both the US and UK went to bed later in the evening and got the least amount of sleep, and also spent less time exercising and outdoors. Subtype one also reports the highest media use and ratings of drug use. Subtype two went to bed early and got above average sleep, but below average exercise, outdoor time, and social media use. Compared to previous subtypes, Subtype three showed relatively divergent patterns across the US and UK, with the US sample showing later than average bedtime and media use, less than average sleep, while in the UK individuals had an early bedtime but greater than average sleep and less media use on average. Subtype four, in the UK only, showed the earliest bedtime, and the greatest amount of sleep, exercise and outdoor time, and the least amount of media use.

One-way ANOVA of subtype by Prior Mood score and ANCOVA of Current Mood factor score, controlling for Prior Mood Score, indicates that these Prior Habits subtypes show different patterns of Mood States over time (Prior Mood State: US $p > 0.05$, UK $p < 0.00001$; Current Mood State: US $p > 0.05$, UK $p > 0.05$). One-way ANOVA of subtype by COVID Worries factor score shows that these Prior Habits subtypes are sensitive to differences in COVID worries in the UK but not US (US $p > 0.05$, UK $p < 0.00001$). The parent report subtypes show subtype one and two with the highest and lowest COVID worries factor scores respectively. We also see that subtype one and two are significantly different in age distribution, with subtype one having the most teenagers and subtype two having the most children under five.

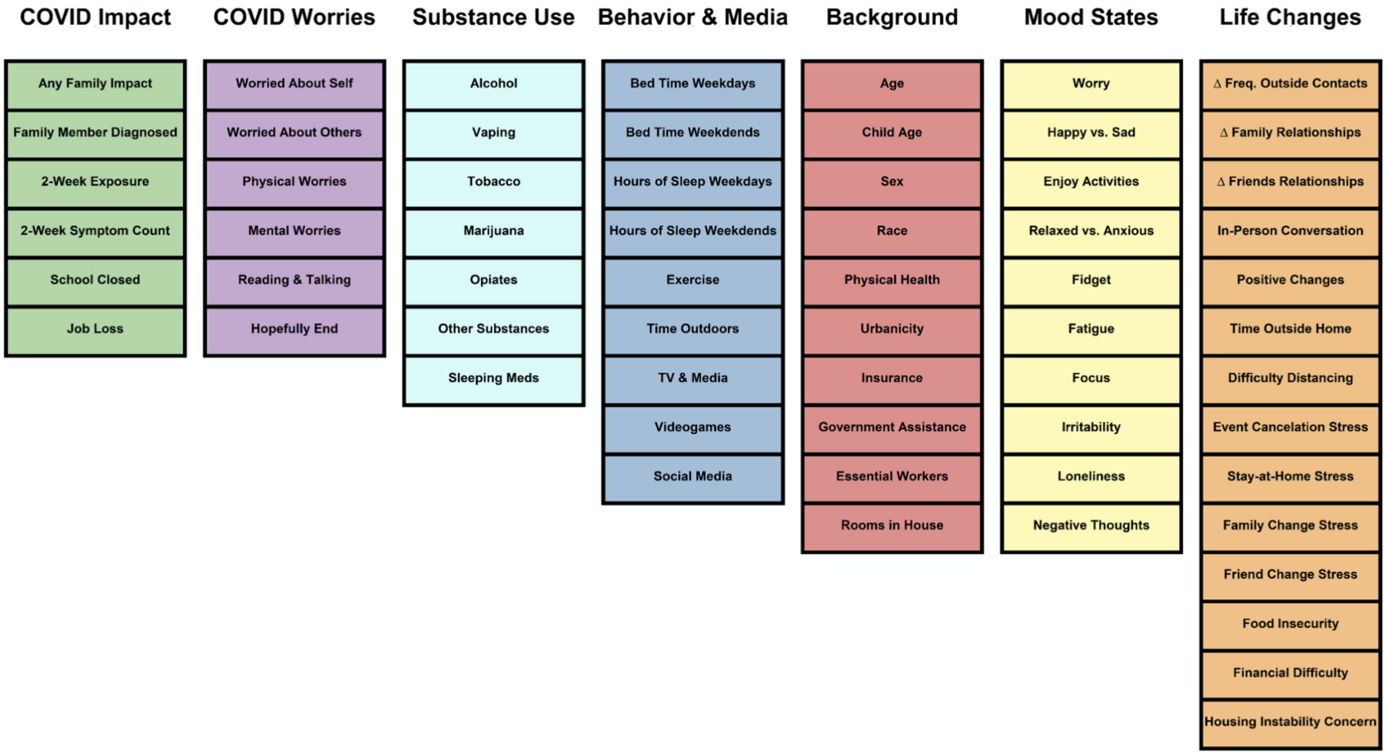

S. Figure 1: Columns contain the variables included in each of the individual categories and colored by category respectively.

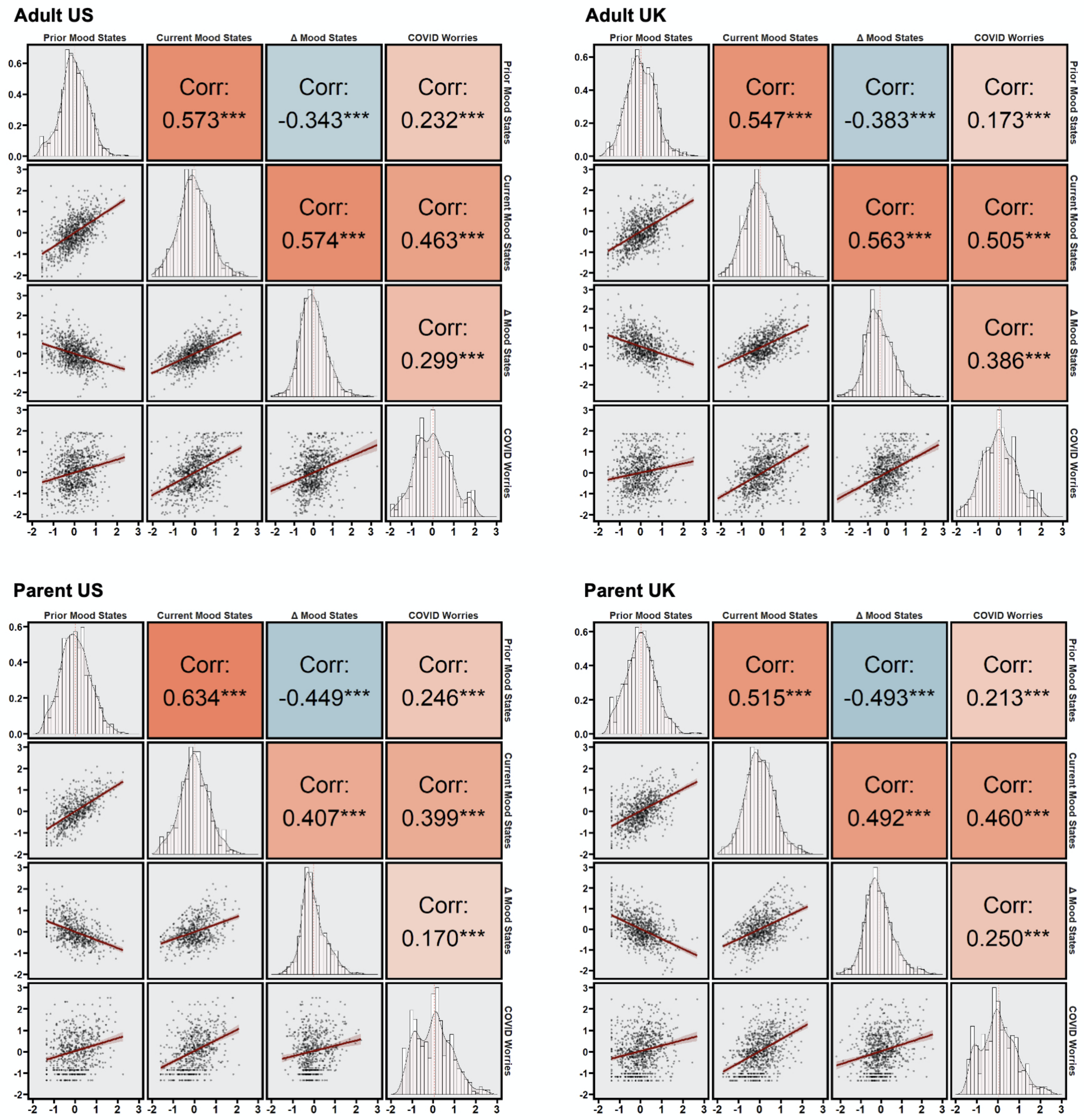

S. Figure 2. Correlations between all factors scores (Prior Mood States, Current Mood States, Δ Mood States, and COVID Worries) across all samples. Lower left panels display matrices of scatter plots of the correlation between the factors. X and Y label values represent a standard loading of -2 at the origin and 3 at the maximum for each of the factors. Corresponding diagonal panels show the histogram distribution of the factor correlations. Pearson correlation coefficient values are presented in the top right panels.

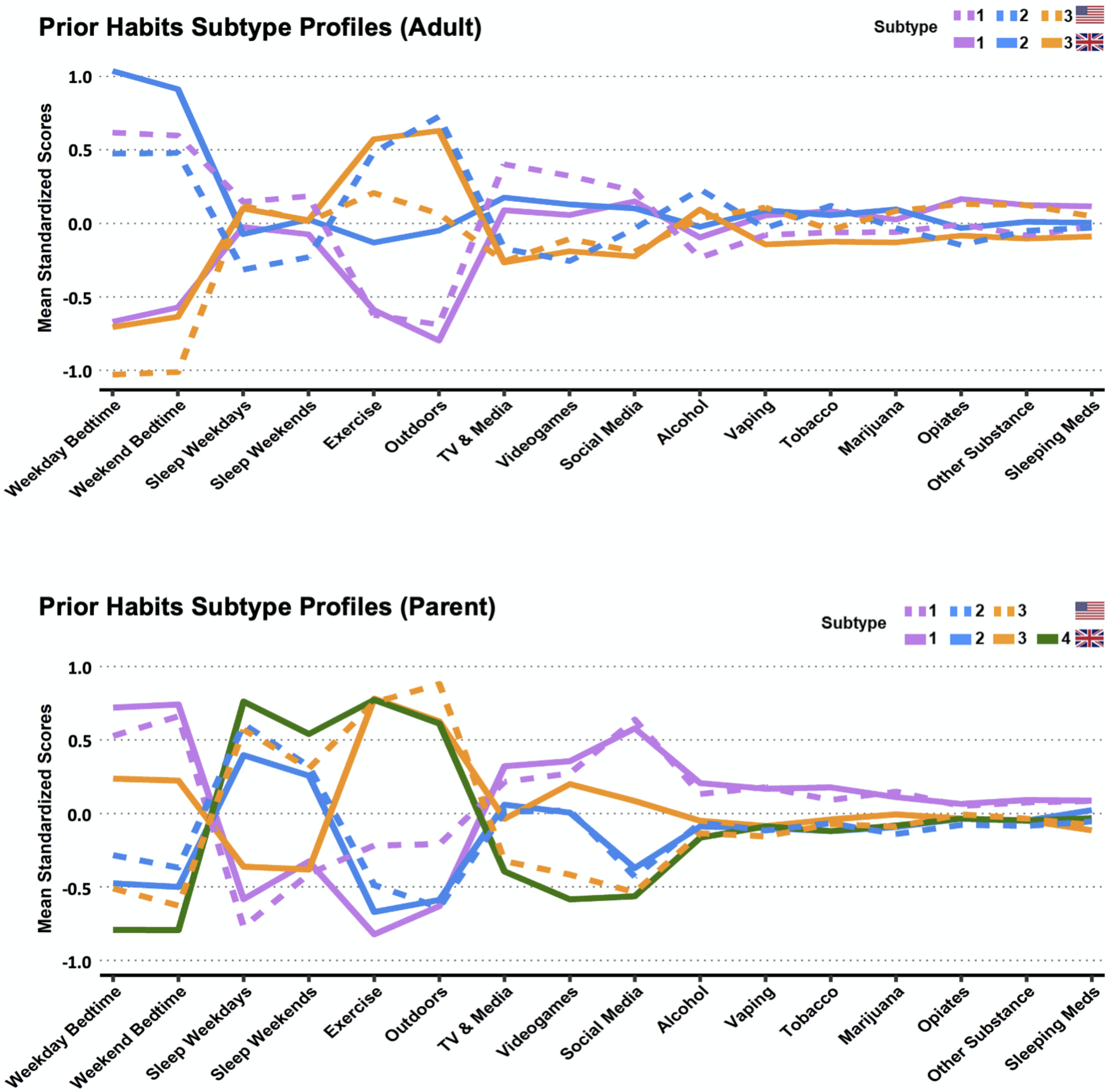

S. Figure 3: Prior Habit Subtype profiles from adult self-reports and parent reports. Mean normalized profile loadings are displayed on the y-axis. US subtypes in solid lines, UK in dashed lines.

	Adult US Prior Habits Subtype 1	Adult US 2	Adult US 3	Adult UK Prior Habits Subtype 1	Adult UK 2	Adult UK 3	Parent US Prior Habits Subtype 1	Parent US 2	Parent US 3	Parent UK Prior Habits Subtype 1	Parent UK 2	Parent UK 3	Parent UK 4
**Factor Scores**													
**COVID Worries** p-value (η^2)	p > 0.05 (0.02)			p < 0.01 (0.01)			p < 0.01 (0.01)			p < 0.0001 (0.03)			
				**		**	** *	**	*	*** * ***	***	*	***
Mean SD	0.02 (0.86)	-0.06 (0.85)	0.05 (0.87)	0.13 (0.87)	-0.01 (0.82)	-0.11 (0.79)	0.22 (0.82)	-0.13 (0.77)	0.01 (0.82)	0.21 (0.81)	-0.13 (0.76)	0.01 (0.81)	-0.11 (0.85)
**Prior Mood States** p-value (η^2)	p < 0.0001 (0.02)			p < 0.0001			p > 0.05 (0.004)			p < 0.0001 (0.06)			
	** ***	**	***	***	**	*** **				*** *** ***	***	***	***
Mean SD	0.13 (0.66)	-0.05 (0.64)	-0.09 (0.59)	0.05 (0.65)	0.11 (0.68)	0.14 (0.62)	0.01 (0.74)	0.05 (0.64)	-0.06 (0.64)	0.24 (0.68)	-0.02 (0.63)	-0.10 (0.71)	-0.17 (0.63)
**Current Mood States** p-value (η^2)	P < 0.0001 (0.17)			p > 0.05 (0.02)			p > 0.05 (0.002)			p > 0.05 (0.008)			
	***	*	*** *										
Mean SD	-0.01 (0.72)	-0.04 (0.73)	0.05 (0.73)	0.05 (0.72)	0.05 (0.76)	-0.07 (0.70)	0.01 (0.69)	-0.05 (0.66)	0.04 (0.67)	0.09 (0.68)	-0.05 (0.67)	-0.03 (0.68)	-0.04 (0.71)
**Background**													
**Sex** p-value (η^2)	p > 0.05 (0.001)			p < 0.05 (0.005)			p > 0.05 (0.01)			p > 0.05 (0.008)			
				**	**								
Male	40.82%	43.54%	44.13%	36.40%	47.66%	44.16%	57.35%	53.45%	47.92%	54.61%	50.76%	46.70%	54.96%
Female	58.23%	56.46%	54.29%	63.60%	52.34%	55.52%	41.94%	45.98%	57.89%	45.05%	49.24%	52.75%	45.04%
**Age** p-value (η^2)	p < 001 (0.04)			p < 0.0001			p > 0.05 (0.02)			p < 0.05 (0.03)			
	***	*	*** *	*** **	*** **	** **				* *	* *	* *	* *
Under 30 %	50.63%	42.44%	34.60%	19.74%	41.87%	28.08%	11.47%	16.67%	9.90%	12.97%	5.30%	14.29%	6.61%
30 – 49 %	28.48%	43.81%	43.81%	55.70%	34.71%	41.32%	81.00%	79.89%	83.85%	79.18%	87.88%	78.57%	87.60%
Over 50 %	20.89%	24.72%	21.59%	24.56%	23.43%	30.60%	7.53%	3.45%	6.25%	7.85%	6.82%	7.14%	5.79%
**Child Age** p-value (η^2)	**			**			p < 0.0001 (0.40)			p < 0.0001 (0.40)			
							*** ***	***	***	* *** ***	* *** ***	*** *** ***	*** *** ***
5 and Under %	**	**	**	**	**	**	5.04%	24.14%	18.23%	5.80%	21.37%	7.69%	40.50%
6 – 13 %	**	**	**	**	**	**	50.72%	66.09%	72.92%	46.76%	73.28%	67.58%	54.96%
14 – 17 %	**	**	**	**	**	**	37.77%	9.77%	8.85%	41.64%	5.34%	21.43%	4.13%
18 and Older %	**	**	**	**	**	**	6.47%	**	**	5.80%	**	3.30%	0.41%
**Race** p-value (η^2)	p < 0.01 (0.04)			p > 0.05 (0.02)			p > 0.05 (0.03)			p > 0.05 (0.01)			
	***	*	*** *										
Asian %	16.46%	17.34%	8.57%	4.82%	8.54%	6.94%	1.08%	4.60%	3.12%	5.12%	4.55%	4.95%	3.72%
Black %	8.54%	8.12%	8.25%	5.26%	4.68%	3.47%	7.89%	4.02%	3.65%	5.80%	5.30%	4.40%	3.31%
Hispanic %	20.25%	14.39%	13.95%	0.88%	2.75%	1.58%	15.77%	12.07%	15.10%	1.71%	0.76%	2.20%	1.65%
Other %	4.75%	5.90%	6.03%	3.95%	6.06%	3.79%	5.73%	4.02%	4.17%	3.41%	1.52%	2.20%	3.31%
White %	50.00%	54.24%	63.49%	85.09%	77.96%	84.23%	69.53%	75.29%	73.96%	83.96%	87.88%	85.26%	88.02%
**Household Density** p-value (η^2)	p < 0.01 (0.01)			p > 0.05 (0.03)			p > 0.05 (0.005)			p > 0.05 (0.03)			
	*		*										
Mean (SD)	5.06 (3.05)	5.53 (3.32)	5.76 (3.02)	5.96 (2.48)	5.59 (2.79)	6.27 (2.78)	5.90 (2.96)	6.56 (3.25)	6.76 (3.39)	5.86 (2.47)	6.60 (2.59)	5.60 (2.55)	6.48 (2.39)
**Rooms in House** p-value (η^2)	p < 0.01 (0.009)			p > 0.05 (0.03)			p < 0.01 (0.01)			p > 0.05 (0.04)			
	*		*				* *	*	*				
Mean (SD)	0.91 (0.56)	0.71 (0.51)	0.63 (0.46)	0.69 (0.56)	0.71 (0.51)	0.63 (0.46)	0.89 (0.53)	0.82 (0.51)	0.81 (0.52)	0.81 (0.53)	0.70 (0.37)	0.88 (0.50)	0.71 (0.47)
**COVID Impact**													
**School Closed** p-value (η^2)	p > 0.05 (0.02)			p < 0.01 (0.03)			p < 0.001 (0.06)			p < 0.01 (0.05)			
				**	** *	*	**	*** **	***	**	*	*	** * *
School Closed but Classes Resumed Online %	26.43%	24.62%	19.75%	6.28%	15.86%	8.09%	74.64%	60.92%	69.93%	47.72%	46.97%	44.51%	32.64%
School Closed but Classes Did Not Close %	0.64%	0.76%	1.59%	3.14%	3.68%	4.21%	16.30%	19.54%	24.61%	45.36%	45.36%	49.54%	57.44%
School Did Not Close %	0.96%	1.89%	3.82%	2.24%	3.12%	3.24%	2.90%	4.60%	1.05%	3.09%	7.58%	2.75%	7.44%
Not Applicable %	71.97%	72.73%	74.84%	88.34%	77.24%	84.47%	6.16%	14.94%	4.71%	4.12%	3.03%	3.30%	2.48%
**Job Loss** p-value (η^2)	p < 0.01 (0.02)			p < 0.05 (0.02)			**			**			
	**		**	*	* *	*							
Job Prior to Pandemic and Still Working %	40.58%	44.36%	48.09%	53.54%	42.58%	25.24%	**	**	**	**	**	**	**
Job Prior to Pandemic and Not Still Working %	24.60%	27.82%	29.30%	24.34%	24.65%	51.12%	**	**	**	**	**	**	**
Did Not Have Job Prior to Pandemic %	34.82%	27.82%	22.61%	22.12%	32.77%	23.64%	**	**	**	**	**	**	**

Subtype 1 | Subtype 2 | Subtype 3 | * p < 0.05 | ** p < 0.01 | *** p < 0.001

S Figure 4. Prior Habits subtypes are indicated by color (Subtype 1, purple; Subtype 2, blue; Subtype 3, orange). Significant ANOVA group differences (COVID Worries, Prior Mood States, and Current Mood States) and Chi-Square group differences (Sex, Age, Child Age, Race, School Closed, and Job Loss) are represented by white asterisks; * *p* < 0.05, ** *p* < 0.01, ****p* < 0.001 and by color according which subtype significant differences were observed.

	US Adult	UK Adult	US Parent	UK Parent
**Missing Data**				
Mean (SD)	0.6 (1.8)	0.5 (1.1)	0.4 (0.9)	0.4 (1.0)
**Range of Missed Items**	0-54	0-17	0-14	0-12
**Time to Completion**				
Mean (SD)	13.9 (13.1)	14.4 (11.5)	14.1 (7.5)	13.9 (20.9)

**Incomplete Observations**				
N (%)	108 (6.0)	43 (2.6)	43 (3.5)	88 (5.4)

S. Table 1. Missing data are summarized. First, the number of items missing on average in each completed survey; Second, the range of missing items across all completed surveys, and third the average time to completion of the surveys (in minutes). Fourth, the total number of incomplete surveys for each sample.

**Life Changes ICC**		**Behavior & Media ICC**			**Substance Use ICC**		
	**Current** Mean (SD)		**Prior** Mean (SD)	**Current** Mean (SD)		**Prior** Mean (SD)	**Current** Mean (SD)
**Positive Change**	0.66 (0.07)	**Bedtime Weekdays**	0.78 (0.06)	0.86 (0.08)	**Alcohol**	0.83 (0.19)	0.66 (0.32)
**Time Outside**	0.66 (0.08)	**Bedtime Weekends**	0.80 (0.07)	0.86 (0.04)	**Vaping**	0.92 (0.11)	0.98 (0.02)
**Contacts Changed**	0.36 (0.12)	**Hours of Sleep Weekdays**	0.80 (0.05)	0.79 (0.08)	**Tobacco**	0.99 (0.01)	0.98 (0.07)
**Family Change**	0.58 (0.06)	**Hours of Sleep Weekends**	0.78 (0.04)	0.77 (0.09)	**Marijuana**	0.98 (0.03)	0.95 (0.05)
**Friends Change**	0.70 (0.13)	**Exercise**	0.76 (0.05)	0.82 (0.07)	**Opiates**	0.80 (0.36)	0.78 (0.37)
**Cancelations Difficulty**	0.64 (0.12)	**Time Outdoors**	0.75 (0.04)	0.82 (0.06)	**Other Substances**	0.95 (0.05)	0.92 (0.09)
**Living Difficulty**	0.65 (0.12)	**TV & Media**	0.62 (0.14)	0.63 (0.12)	**Sleeping Meds**	0.86 (0.16)	0.82 (0.12)
**In-Person Conversation**	0.84 (0.21)	**Social Media**	0.82 (0.05)	0.85 (0.04)			
**Restriction Stress**	0.68 (0.08)	**Video Game**	0.84 (0.04)	0.86 (0.03)			
**Distancing Difficulty**	0.48 (0.13)						
**Family Change Stress**	0.60 (0.09)						
**Friends Change Stress**	0.62 (0.08)						
**Financial Difficulty**	0.78 (0.06)						

S Table 2. Intraclass Correlation Coefficient (ICC) mean and standard deviation for Behavior & Media, Life Changes, and Substance Use variables.

	**Parent US**			**Parent UK**		
	**Mean (SD)**			**Mean (SD)**		
	**Covid Worries**	**Prior Mood States**	**Current Mood States**	**Covid Worries**	**Prior Mood States**	**Current Mood States**
**Population**	0.04 (0.83)	0.01 (0.69)	0.004 (0.67)	0.03 (0.83)	0.007 (0.69)	0.004 (0.68)

**Sex**						
Male	0.03 (0.85)	0.05 (0.67)	0.01 (0.69)	0.02 (0.84)	0.03 (0.69)	0.04 (0.67)
Female	0.05 (0.81)	-0.04 (0.70)	-0.002 (0.64)	0.03 (0.81)	-0.02 (0.68)	-0.03 (0.70)
**Age**				**		**
Under 30	0.02 (0.95)	-0.07 (0.64)	-0.006 (0.73)	0.23 (0.86)	0.08 (0.69)	0.24 (0.70)
30 - 49	0.03 (0.83)	0.03 (0.69)	0.02 (0.67)	-0.01 (0.81)	-0.01 (0.68)	-0.02 (0.67)
50 and older	0.12 (0.63)	-0.08 (0.72)	-0.14 (0.54)	0.21 (0.81)	0.16 (0.73)	-0.002 (0.76)
**Child Age**	***	**		***	***	
5 and Under	-0.55 (0.65)	-.20 (0.53)	-0.13 (0.61)	-0.24 (0.87)	-0.21 (0.65)	-0.07 (0.70)
6 - 13	0.13 (0.80)	0.06 (0.68)	0.04 (0.66)	0.06 (0.84)	0.02 (0.67)	0.04 (0.66)
13 - 17	0.13 (0.84)	-0.02 (0.76)	-0.02 (0.71)	0.17 (0.67)	0.14 (0.70)	0.02 (0.64)
18 and Over	0.31 (0.80)	0.33 (0.63)	0.08 (0.73)	0.24 (0.91)	0.22 (0.79)	-0.01 (0.74)
**Race**					*	**
Asian	0.06 (0.86)	0.01 (0.58)	-0.20 (0.48)	0.08 (0.93)	-0.18 (0.69)	-0.20 (0.60)
Black	0.003 (0.94)	-0.10 (0.79)	-0.19 (0.78)	-0.02 (1.07)	-0.27 (0.71)	-0.36 (0.75)
Hispanic	0.12 (0.82)	-0.03 (0.71)	0.08 (0.64)	-0.14 (0.86)	0.11 (0.86)	-0.13 (0.89)
Other	0.38 (1.15)	0.18 (0.76)	-0.01 (0.80)	0.31 (0.96)	0.12 (0.57)	0.07 (0.62)
White	-0.00006 (0.80)	0.02 (0.67)	0.01 (0.66)	0.03 (0.80)	0.03 (0.68)	0.04 (0.67)
**School Closed**	***					
School Closed but Classed Resumed Online	0.13 (0.81)	0.01 (0.68)	0.02 (0.66)	0.07 (0.80)	-0.02 (0.67)	-0.05 (0.67)
School Closed but Classes Did Not Resume	-0.09 (0.85)	0.05 (0.73)	0.05 (0.67)	-0.004 (0.85)	0.01 (0.70)	0.04 (0.68)
School Did Not Close	-0.18 (0.92)	-0.07 (0.60)	-0.31 (0.56)	-0.01 (0.69)	0.07 (0.49)	0.06 (0.65)
Not Applicable	-0.32 (0.79)	0.06 (0.70)	-0.09 (0.71)	0.03 (0.80)	0.31 (0.84)	0.12 (0.86)
**Essential Worker in Family**						
No	0.09 (0.83)	0.03 (0.70)	0.01 (0.67)	0.02 (0.84)	0.12 (0.68)	-0.01 (0.68)
Yes	-0.05 (0.84)	-0.05 (0.66)	-0.03 (0.66)	0.04 (0.80)	0.02 (0.69)	0.05 (0.71)
Yes, works in COVID facility	-0.05 (0.84)	0.14 (0.69)	0.23 (0.83)	0.07 (0.92)	-0.05 (0.66)	-0.05 (0.66)
**Any Family Impact**	***	**	***	*		***

No	-0.03 (0.80)	-0.03 (0.66)	-0.07 (0.64)	-0.02 (0.83)	0.01 (0.68)	-0.003 (0.68)
Yes	0.22 (0.90)	0.13 (0.74)	0.22 (0.71)	0.32 (0.74)	0.08 (0.79)	0.20 (0.69)
**Family Member Diagnosed**				*		*
No	0.03 (0.82)	0.01 (0.69)	-0.001 (0.66)	0.02 (0.83)	0.01 (0.68)	-0.003 (0.68)
Yes	0.24 (1.15)	0.29 (0.79)	0.23 (0.83)	0.32 (0.74)	0.08 (0.79)	0.20 (0.69)
**2-Week COVID Exposure**	***	***	***			
None	0.01 (0.81)	-0.01 (0.68)	-0.02 (0.65)	0.03 (0.83)	0.001 (0.69)	0.01 (0.67)
Exposure to person with symptoms	0.39 (1.81)	0.40 (0.63)	0.36 (0.81)	0.05 (0.80)	0.09 (0.67)	-0.04 (0.80)
Exposure to person with diagnosis	0.90 (0.80)	0.67 (0.46)	0.69 (0.66)	0.07 (0.75)	0.07 (0.72)	0.18 (0.61)
**2-Week Symptom Count**	***	***	***			**
None	-0.03 (0.81)	-0.04 (0.68)	-0.06 (0.65)	0.01 (0.82)	-0.01 (0.68)	-0.02 (0.67)
One	0.34 (0.80)	0.30 (0.66)	0.25 (0.63)	0.10 (0.79)	0.09 (0.69)	0.11 (0.65)
Two	0.67 (0.81)	0.31 (0.80)	0.63 (0.55)	0.03 (0.85)	-0.05 (0.60)	-0.09 (0.69)
Three or more	0.36 (1.14)	0.40 (0.54)	0.42 (0.73)	0.34 (0.89)	0.22 (0.76)	0.40 (0.82)

S Table 3: Parent report overall mean and SD of factor scores (COVID Worries and Mood States) followed by mean and SD by demographic group and COVID-related characteristics. Significant ANOVA demographic group differences are represented by asterisks; * $p < 0.05$, ** $p < 0.01$, ***$p < 0.001$.

	**Adult US**	**Adult UK**	**Parent US**	**Parent UK**
**Subtype Membership**	$\chi^2 = 31.03$; $p < 0.00001$	$\chi^2 = 30.21$; $p < 0.00001$	$\chi^2 = 58.25$; $p < 0.00001$	$\chi^2 = 15.42$; $p < 0.005$

S. Table 4. Chi-Square tests were conducted between the Prior Habits and Life Changes subtypes to assess whether Life Changes subtype membership differed significantly according to Prior Habit subtypes. P values < 0.05 indicate significant differences in membership between Prior Habits and Life Changes subtypes.

	**Adult US**	**Adult UK**	**Parent US**	**Parent UK**
R^2	0.512	0.496	0.566	0.483

S. Table 5. Out of sample performance of linear models in predicting current mood. Linear models used the top four variables from the random forest. For Adult self report this included Life Changes subtype, COVID Worries score, Prior Mood States, and age as well as interactions of COVID Worries and Age, Prior Mood States and

Age, Life Changes subtype and Age, Prior Mood States and Life Changes subtype, COVID Worries and Life Changes Subtype, and COVID Worries and Prior Mood States. For the Parent report the same variables and interactions were included, but physical health was used instead of Age.

	US		UK	
	In Sample	Out of Sample	In Sample	Out of Sample
	Adult Report			
COVID Worries (Correlation)	p < 0.00001, (0.46)	p < 0.00001, (0.46)	p < 0.00001, (0.51)	p < 0.00001, (0.50)
Prior Mood States (Correlation)	p < 0.00001, (0.57)	p < 0.00001, (0.58)	p < 0.00001, (0.55)	p < 0.00001, (0.54)
Lifestyle Changes Subtypes	p < 0.00001	p < 0.00001	p < 0.00001	p < 0.00001
Age	p < 0.00001	p < 0.00001	p < 0.00001	p < 0.00001
	Parent Report			
COVID Worries (Correlation)	p < 0.00001, (0.40)	p < .00005, (0.44)	p < 0.00001, (0.46)	p < 0.00001, (0.47)
Prior Mood States (Correlation)	p < 0.00001, (0.64)	p < 0.00001, (0.61)	p < 0.00001, (0.51)	p < 0.00001, (0.53)
Lifestyle Changes Subtypes	p < 0.00001	p < 0.00001	p < 0.00001	p < 0.00001
Physical Health	p < 0.00001	p < 0.00001	p < 0.00001	p < 0.00001

S. Table 6. Random Forest tests identified the above five variables (COVID Worries, Prior Mood States, Life Changes Subtypes, Age, and Physical Health) to be the most important for predicting Current Mood States. The relationship between COVID Worries, Prior Mood States and Current Mood States was tested with Pearson correlations in and out of sample. ANOVA was used to test the difference in Current Mood States with Age (Adult Report) and parent-rated Physical Health (Parent Report) out of sample. P values < 0.05 indicate significant differences in membership between Prior Habits and Life Changes subtypes. Correlation values are provided next the p value for COVID Worries and Prior Mood States.
